# A prospective study on the usefulness of high-resolution intraoperative infrared thermography in intracranial tumors

**DOI:** 10.3389/fsurg.2024.1386722

**Published:** 2024-06-12

**Authors:** Diego Rodrigues Menezes, Lázaro de Lima, Raíssa Mansilla, Aura Conci, Fernanda Rueda, Luis Guilhermo Coca Velarde, José Alberto Landeiro, Marcus André Acioly

**Affiliations:** ^1^ Division of Neurosurgery, Fluminense Federal University, Rio de Janeiro, Brazil; ^2^ Postgraduation Program in Neurology, Federal University of the State of Rio de Janeiro (UNIRIO), Rio de Janeiro, Brazil; ^3^ Department of Computer Science, Fluminense Federal University, Rio de Janeiro, Brazil; ^4^ Division of Radiology, Fluminense Federal University, Rio de Janeiro, Brazil; ^5^ Department of Statistics, Fluminense Federal University, Rio de Janeiro, Brazil; ^6^ Division of Neurosurgery, Federal University of Rio de Janeiro (UFRJ), Rio de Janeiro, Brazil

**Keywords:** image-guided surgery, thermal imaging, infrared imaging, brain tumor, thermography, brain temperature

## Abstract

**Introduction:**

Infrared thermography (IT) is a non-invasive real-time imaging technique with potential application in different areas of neurosurgery. Despite technological advances in the field, intraoperative IT (IIT) has been an underestimated tool with scarce reports on its usefulness during intracranial tumor resection. We aimed to evaluate the usefulness of high-resolution IIT with static and dynamic thermographic maps for transdural lesion localization, and diagnosis, to assess the extent of resection, and the occurrence of perioperative acute ischemia.

**Methods:**

In a prospective study, 15 patients affected by intracranial tumors (six gliomas, four meningiomas, and five brain metastases) were examined with a high-resolution thermographic camera after craniotomy, after dural opening, and at the end of tumor resection.

**Results:**

Tumors were transdurally located with 93.3% sensitivity and 100% specificity (*p* < 0.00001), as well as cortical arteries and veins. Gliomas were consistently hypothermic, while metastases and meningiomas exhibited highly variable thermographic maps on static (*p* = 0.055) and dynamic (*p* = 0.015) imaging. Residual tumors revealed non-specific static but characteristic dynamic thermographic maps. Ischemic injuries were significantly hypothermic (*p* < 0.001).

**Conclusions:**

High-resolution IIT is a non-invasive alternative intraoperative imaging method for lesion localization, diagnosis, assessing the extent of tumor resection, and identifying acute ischemia changes with static and dynamic thermographic maps.

## 1 Introduction

Maximal safe resection is the well-recognized first step of a multimodal therapy algorithm for gliomas and brain metastases. For years, the extent of resection was a debatable issue in brain tumors, but we have come to the point that gross total resection is an essential step in managing these patients being one of the most important predictors of overall survival for malignant gliomas, low-grade gliomas, and brain metastases (1–7).

To achieve radical tumor removal, several tools were developed or sophisticated to provide better visualization of tumor tissue or to identify eloquent neural networks guiding resection to the functional boundaries. Three major methods are generally explored in the literature, namely based on imaging [neuronavigation, intraoperative magnetic resonance imaging (iMRI), and intraoperative ultrasound (iUS)] (1, 2, 8, 9), based on fluorescence (5-ALA, and sodium fluorescein) (10, 11), and based on functional mapping (7, 12, 13).

Conversely, each method presents specific limitations that involve brain shift for neuronavigation, high costs, and technical constraints (such as infection control, artifacts, and equipment compatibility) for iMRI and iUS (14–17), low contrast index in low-grade gliomas, tissue overlapping and allergy for fluorescence-guided surgery (18–20), as well as preoperative severe neurological deficits, medical contraindications, and inter-individual anatomo-functional variability for brain mapping (7, 21).

In this context, intraoperative infrared thermography (IIT) emerges as a potential alternative for locating tumors and tumor margins during surgery. IIT has been an underestimated tool with few reports on its usefulness during intracranial tumor resection (22–28). While infrared thermography is a technology under constant refinement, we then conducted a prospective study to evaluate the usefulness of IIT with a high-resolution thermographic camera for transdural lesion-localization, diagnosis, to assess the extent of resection, and the occurrence of perioperative acute ischemia.

## 2 Materials and methods

### 2.1 Study design

This is a prospective and cross-sectional observational study with pre-, intra-, and postoperative clinical and imaging data collection. This study followed the guidelines of the Strengthening the Reporting of Observational Studies in Epidemiology (STROBE) statement for writing observational studies (29) and was approved by the local ethics committee (CAAE 32871020.4.0000.5258/2019). Informed consent was obtained from all patients. Patients with a histologically confirmed diagnosis of primary (gliomas and meningiomas) and secondary brain tumors (brain metastases) aged over 18 years with surgical indication were included. Patients with deep-positioned tumors (skull base, insular or basal ganglia, and thalamus), patients with an inconclusive histological diagnosis, and patients with recurrent brain tumors or previously irradiated brain tumors were excluded.

### 2.2 Data collection and outcome measurement

Patient age, gender, the occurrence of tumor recurrence/relapse, intraoperative blood pressure, surgical position, type of anesthesia, as well as patient and operating room temperatures, and ambient humidity information, were collected. All patients had their temperature continuously monitored by an esophageal thermometer attached to the anesthesia station. Neuronavigation, iUS, and fluorescence-guided surgery (sodium fluorescein) were not routinely used. Surgeries were performed under general or local anesthesia for intraoperative brain mapping. The decision regarding stopping surgical resection was made by the surgical team, regardless of the findings of the thermographic maps.

Thermographic imaging was performed on a FLIR SC620 camera (FLIR systems, USA), which provides high-spatial resolution (640 × 480 pixels) with a thermal sensitivity of 0.02 °C. To avoid movement artifacts, the camera was placed on a tripod positioned 30 cm–50 cm from the surgical site. Surgical lights were redirected from the surgical field for infrared measurements to avoid tissue heating.

**Figure 1:**
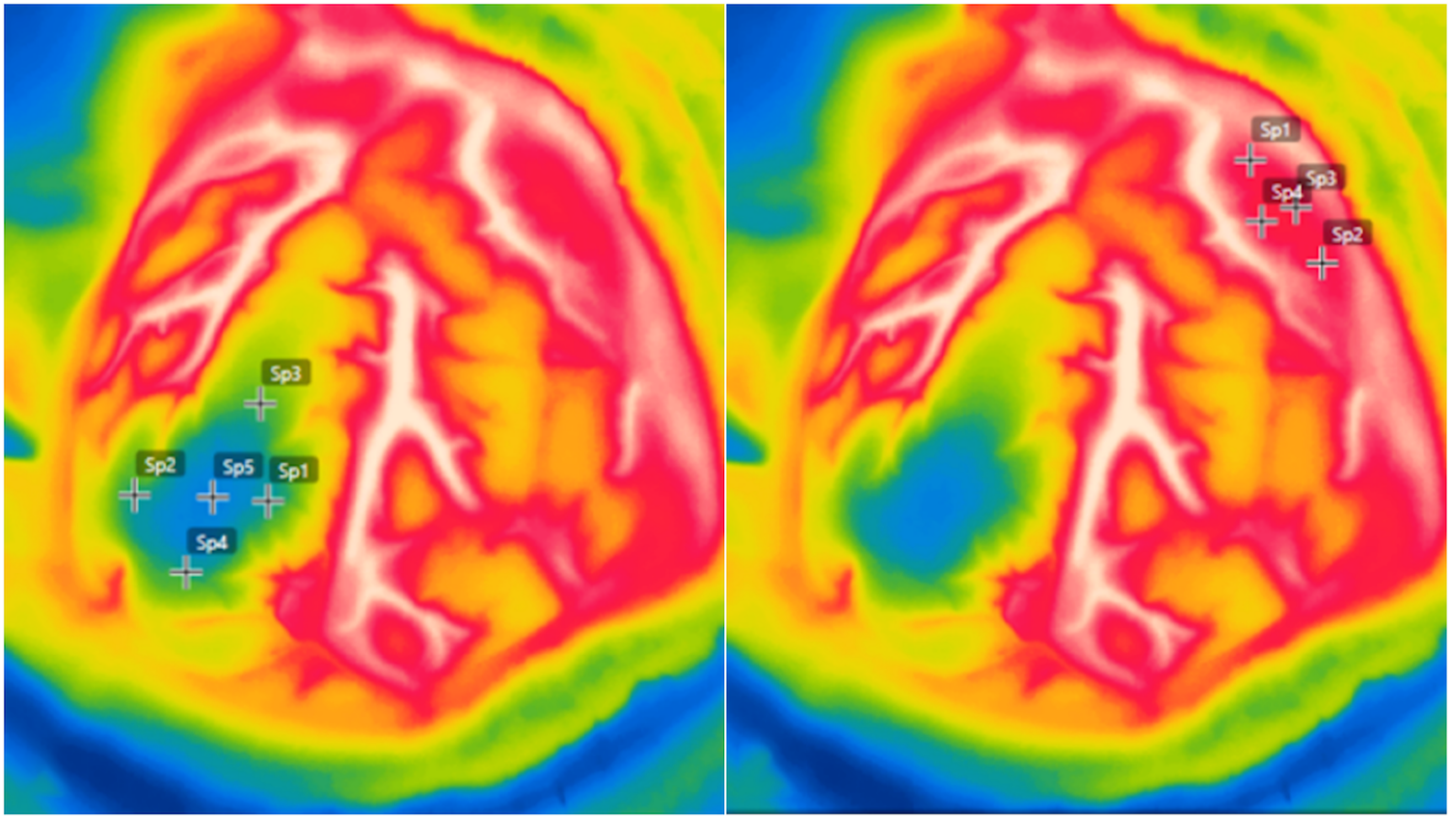
Intraoperative high-resolution infrared thermography imaging demonstrating temperature measurements in previously established locations in the healthy cortex at craniotomy margins (left), and tumoral area over the tumor or adjacent cortex (right). Anterior [A-Spot], posterior [P-Spot], medial [M-Spot], lateral [L-Spot], and central [C-Spot].

Background static and sequential digital images were obtained (60 images per 1-min trial, 3 trials for patient) during irrigation-induced hypothermia of the exposed dura mater or cortex after the instillation of saline solution at room temperature (cold challenge test). In this case, imaging acquisition started approximately 10 s before and ended 1 min after the end of its application. A sudden temperature drop was identified after saline instillation, thereby generating thermal recovery curves of the exposed tissues (24). Image acquisition was performed before durotomy, after durotomy, and at the end of the tumor resection (as indicated by the surgical team) and did not exceed 10 min in any case. Infrared imaging was analyzed postoperatively with the aid of the FLIR Tools® software (FLIR systems, USA) in previously established locations (Figure 1), namely:

• Healthy cortex (control; at craniotomy margins, away from the tumor)—anterior (A-Spot), posterior (P-Spot), medial (M-Spot), and lateral (L-Spot);
• Tumor or adjacent cortex—anterior (A-Spot), posterior (P-Spot), medial (M-Spot), lateral (L-Spot), and central (C-Spot);
• Tumor bed—anterior (A-Spot), posterior (P-Spot), medial (M-Spot), lateral (L-Spot), and central (C-Spot).

Preoperative T2-weighted FLAIR (fluid-attenuated inversion recovery), T1-weighted gadolinium-enhanced, and diffusion-weighted sequences were performed on a Siemens® 1.5T scanner (Erlangen, Germany). Tumor location, depth (distance between the cortical surface and most superficial tumor location; cortical or subcortical location), and tumor dimensions were determined. Postoperatively, patients underwent imaging within 72 h (computed tomography and/or MRI). Postoperative images were analyzed using Radiant® software [Medixant. RadiAnt DICOM Viewer (Software). Version 2020.1. 9 March 2020. URL: https://www.radiantviewer.com] by two different investigators, a neurosurgeon (DRM) and an experienced neuroradiologist (FR), who was blinded for intraoperative findings and thermographic evaluations. Both investigators determined the extent of resection, the location of potential residual tumor (cavity walls and/or tumor bed), as well as the assessment of acute ischemia.

### 2.3 Statistical analyzes

Descriptive analyses were performed using summaries of numerical variables (mean and percentile) and graphs (thermal recovery curve). After dural opening and full exposure of the tumor, intraoperative inspection was taken as the gold standard for diagnostic accuracy of IIT. Then, a 2 × 2 contingency table was created to calculate IIT sensitivity and specificity for tumor location and the significance of the proportion was assessed by the *χ*^2^ test. To analyze the variation of the thermographic maps at different surgical steps, the Friedman test was used. The significance level was set at *p* < 0.05. If Friedman's analysis demonstrated statistical significance, the Wilcoxon test was performed and adjusted for multiple comparisons with a Bonferroni correction, which is the level of confidence (*p* = 0.05) divided by the number of comparisons, in this study three. This correction results in a level of confidence of *p* = 0.017 to be considered statistically significant. The paired Wilcoxon test was used to compare the thermographic maps before and after the cold challenge test, while the Mann–Whitney test was used to compare the thermographic maps of the tumor and the healthy cortex. All analyses were performed using the R 4.0.0 software (http://www.r-project.org/).

## 3 Results

### 3.1 Descriptive data

Fifteen patients comprised the study group, 8 (53.3%) were female with a mean age of 59.4 years (range, 45–73 years). The mean esophageal temperature during surgical procedures was 35.4 °C (range, 33.7 °C–36.7 °C), while the mean operating room temperature was 22.5 °C (range, 20.5 °C–24.6 °C). Fourteen patients were operated on under general anesthesia and one patient underwent awake surgery for brain mapping. Tumors generally affect cortical frontal and parietal lobes on the left side. Subcortical tumors averaged 1.2 cm in depth (range, 0.25 cm–2 cm). Table 1 illustrates the demographics.

**Table 1:** Patient demographics.

Patient	Age (year)/gender	Side	Location	Final pathology
1	51/M	L	SC	glioblastoma (NOS grade IV)
2	53/F	R	SC	glioblastoma (NOS grade IV)
3	64/M	L	SC	glioblastoma (NOS grade IV)
4	45/F	R	C	oligodendroglioma (grade II)
5	66/M	L	SC	glioblastoma (NOS grade IV)
6	66/M	L	C	glioblastoma (NOS grade IV)
7	47/F	L	C	angiomatous meningioma (grade II)
8	62/F	R	C	meningothelial meningioma (grade I)
9	63/F	R	C	meningothelial meningioma (grade I)
10	72/F	L	C	atypical meningioma (grade II)
11	62/F	R	C	Lung AC
12	52/M	L	SC	Lung AC
13	57/F	R	C	Lung AC
14	73/M	L	C	Lung AC
15	59/M	L	SC	Lung AC

AC, adenocarcinoma; C, cortical; F, female; L, left; M, male; NOS, not otherwise specified; R, right; SC, subcortical.

### 3.2 Intraoperative infrared thermography for transdural lesion-localization

**Figure 2:**
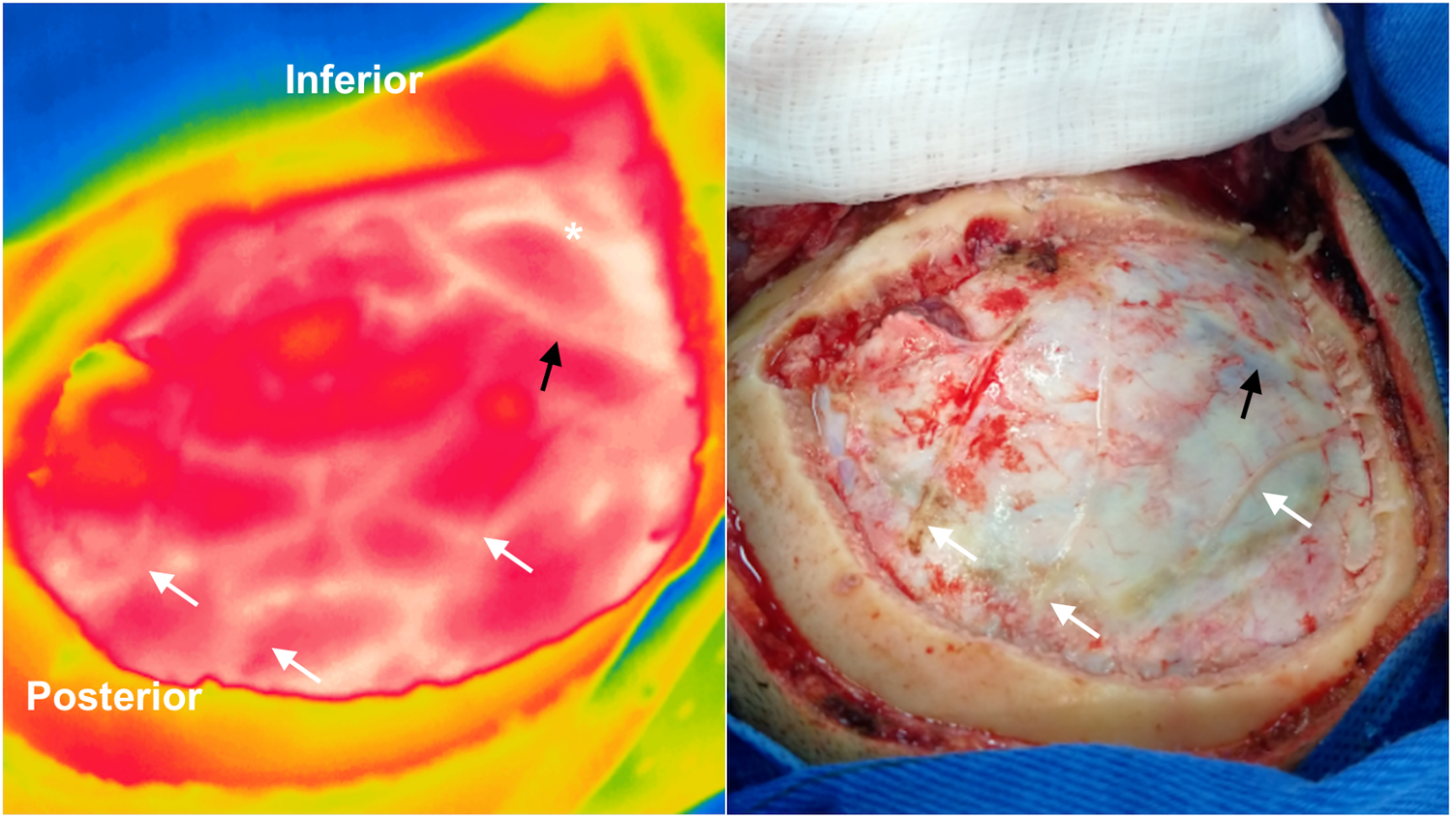
Intraoperative infrared imaging of a left temporal glioblastoma WHO grade IV. A visible light image (right) is provided for comparison. Dural (white arrows) and cortical vessels are superimposed on thermographic imaging after craniotomy. The Labbé vein (black arrows) and cortical arteries (asterisk) can be identified through the dura mater with infrared imaging. In this case, the tumoral area appears hypothermic in the central region of the craniotomy (yellow area). Note the temperature difference between cortical arteries and veins.

Intraoperative thermographic images of the exposed dura mater revealed a mixed vascular pattern of meningeal vessels, veins, and cortical arteries (Figure 2). The temperature difference between vessels, healthy cortex, and intracranial tumors or adjacent cortex was evident. Tumor location was adequately observed in 93.3% of patients through the exposed dura mater (sensitivity 93.3%, specificity 100%, *p* < 0.00001). In three patients, iUS corroborated the thermographic findings regarding lesion localization.

Static thermographic maps of the healthy cortex and tumors immediately after craniotomy showed great variation [mean of 31.9 °C (range, 28.8–34 °C); and mean of 29.7 °C (range, 26.4–33.5 °C), respectively]. Tumors were consistently hypothermic in comparison to the healthy cortex with a mean temperature difference of 2.2 °C (*p* = 0.014) across the exposed dura mater. The coolest site in the tumoral region was the C-Spot (29.4 °C; range, 25–34.1 °C). The cold challenge test significantly decreased the mean temperature both in the healthy cortex and tumors [mean of 30.8 °C (range, 27.2–34.5 °C; *p* = 0.031); mean of 27.8 °C (range, 25–32.6 °C; *p* = 0.006); respectively]. Such exacerbation of absolute temperature differences increased from 2.2 °C to 3 °C, thereby facilitating the identification of tumoral areas. The subcortical location did not interfere with the recognition of tumors up to 2 cm in depth.

### 3.3 Temperatures of the healthy cortex and intracranial tumors before tumor resection

Cortical and tumor area temperatures recovered fast during dural opening reaching baseline values similar to the exposed dura mater [mean of 31.9 °C (*p* = 0.865), and 30.3 °C (*p* = 0.912), respectively]. On the other hand, visualization of cortical vessels was facilitated after dura mater retraction due to increased brightness. Intraparenchymal tumors or the cortex overlying the tumors, in the case of subcortical tumors, were on average 1.6 °C cooler than normal parenchyma (*p* = 0.048). Knowledge of tumor location and vascular relations allowed tumor resection or corticectomy to be precisely planned in all cases.

### 3.4 Temperature gradients of intracranial tumors by histological subtype

**Figure 3:**
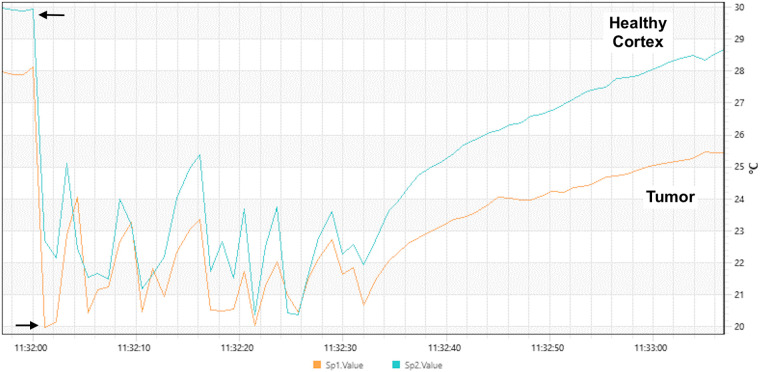
Temperature profiles were obtained from more than 100 images during saline irrigation. Arrows demonstrate the beginning and the end of irrigation. After evaporative cooling, thermal recovery curves were different between the healthy cortex (blue line in the graph) and tumor area (orange line in the graph). Note that the temperature recovery is slower in the tumoral area lesion and reaches a significantly lower temperature compared to the healthy cortex.

Tumors of the glial lineage and brain metastases were primarily hypothermic (mean of 1.8 °C, and 3.5 °C, respectively), while meningiomas were hyperthermic (1.2 °C). The analysis of the C-Spot temperatures significantly elucidated histological subtypes (*p* = 0.055), as well as the cold challenge test (*p* = 0.015) (Figure 3). Interestingly, two patients with meningiomas developed the hottest thermographic maps after dural opening, due to intense bipolar coagulation for hemostasis of dural feeders (2.9 °C and 12.9 °C).

### 3.5 Thermographic profile after tumor resection and correlation with postoperative imaging

Once the resection was deemed complete by the surgical team, the thermographic profiles of the tumor bed were measured. In two glioblastomas, the estimation of the extent of resection was done by fluorescence-guided tissue staining (fluorescein sodium). Static thermographic maps of the tumor bed revealed an average increase of 2.8 °C in gliomas (range, 31.2–34.3 °C), and of 4 °C in brain metastases (range, 30.3–34 °C). Glioma tumor resection beds had the highest absolute temperatures when compared to other histological subtypes. In general, the cold challenge test reduced the mean temperature of the tumor bed to 30.5 °C (range, 27.4–33.8 °C), but did not reach statistical significance.

**Figure 4:**
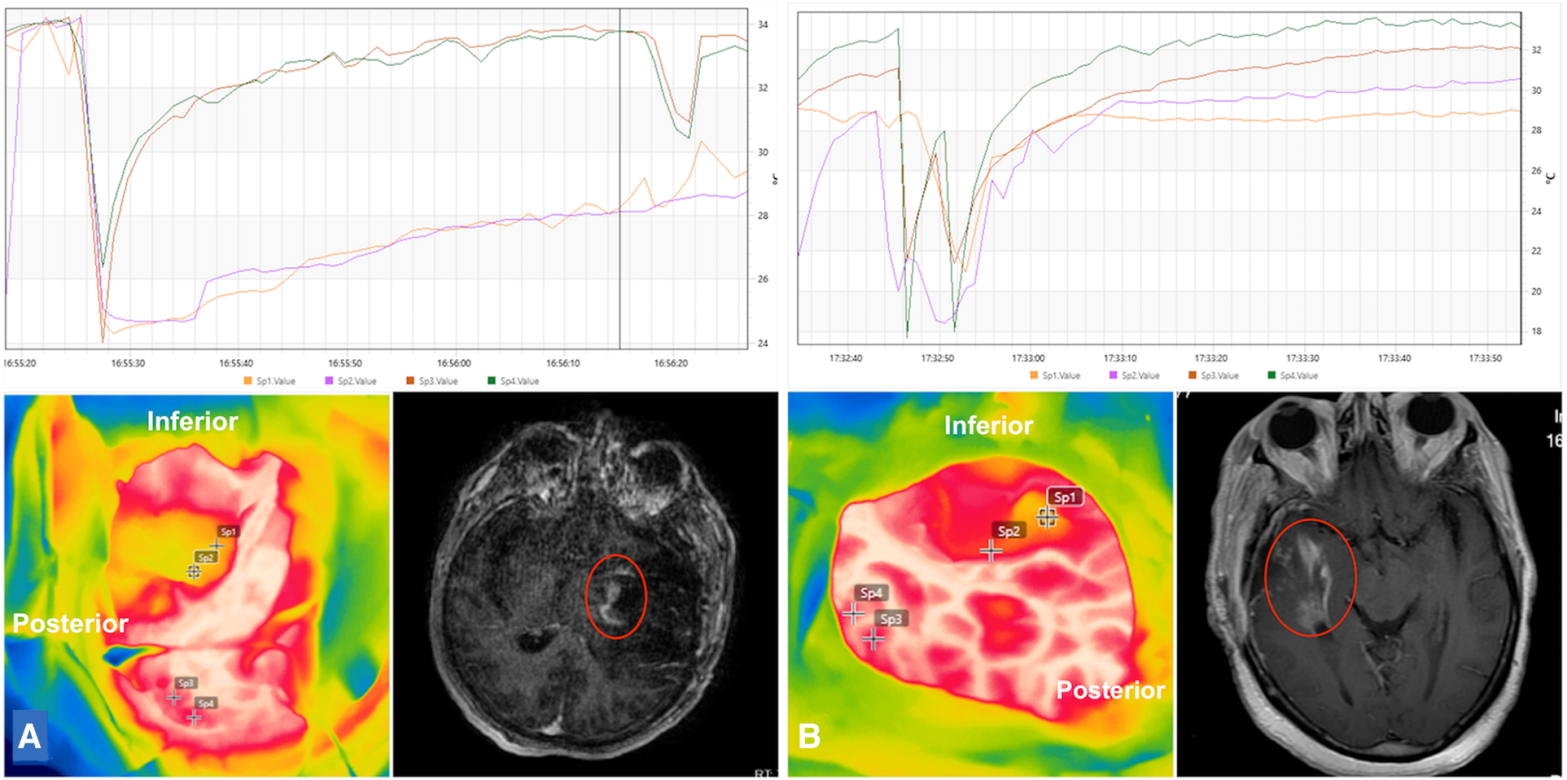
Intraoperative infrared thermographic imaging of the tumor beds of two temporal glioblastomas (**A,B**; lower left). Suspected areas of delayed temperature recovery curves were marked and correlated to postoperative imaging. Residual tumoral areas thermographic profiles demonstrated similar thermal recovery curves of tumoral areas obtained before tumor resection, namely steady and slow temperature recovery and lower final temperature (orange and purple lines in graphs) in comparison to the healthy cortex (dark orange and green lines in graphs) (**A,B**; upper row). Postoperative gadolinium-enhanced T1-weighted images demonstrated residual tumors at the tumor beds (**A,B**; lower right).

Ten patients underwent postoperative MR imaging to estimate the degree of tumor resection and to identify acute ischemia. From these, 50 thermographic measurements were done from the tumor bed. Gross total resection was achieved in 60% of the patients (*n* = 6). In four patients (three glioblastomas and 1 metastasis), the resection was deemed near-total or subtotal. Nine areas (18%) of residual tumors on imaging had their thermographic maps retrospectively evaluated. Residual glioblastomas were isothermic (31.9 °C), while residual metastasis was hypothermic in comparison to the surrounding healthy cortex (28.6 °C and 30 °C, respectively). Besides static thermographic imaging, we also evaluated temperature recovery curves after the cold challenge test, which revealed a characteristic delay in the recovery of the thermographic profile at residual tumor spots, when compared to the healthy cortex (Figure 4). In addition, the final absolute temperature was reduced.

**Figure 5:**
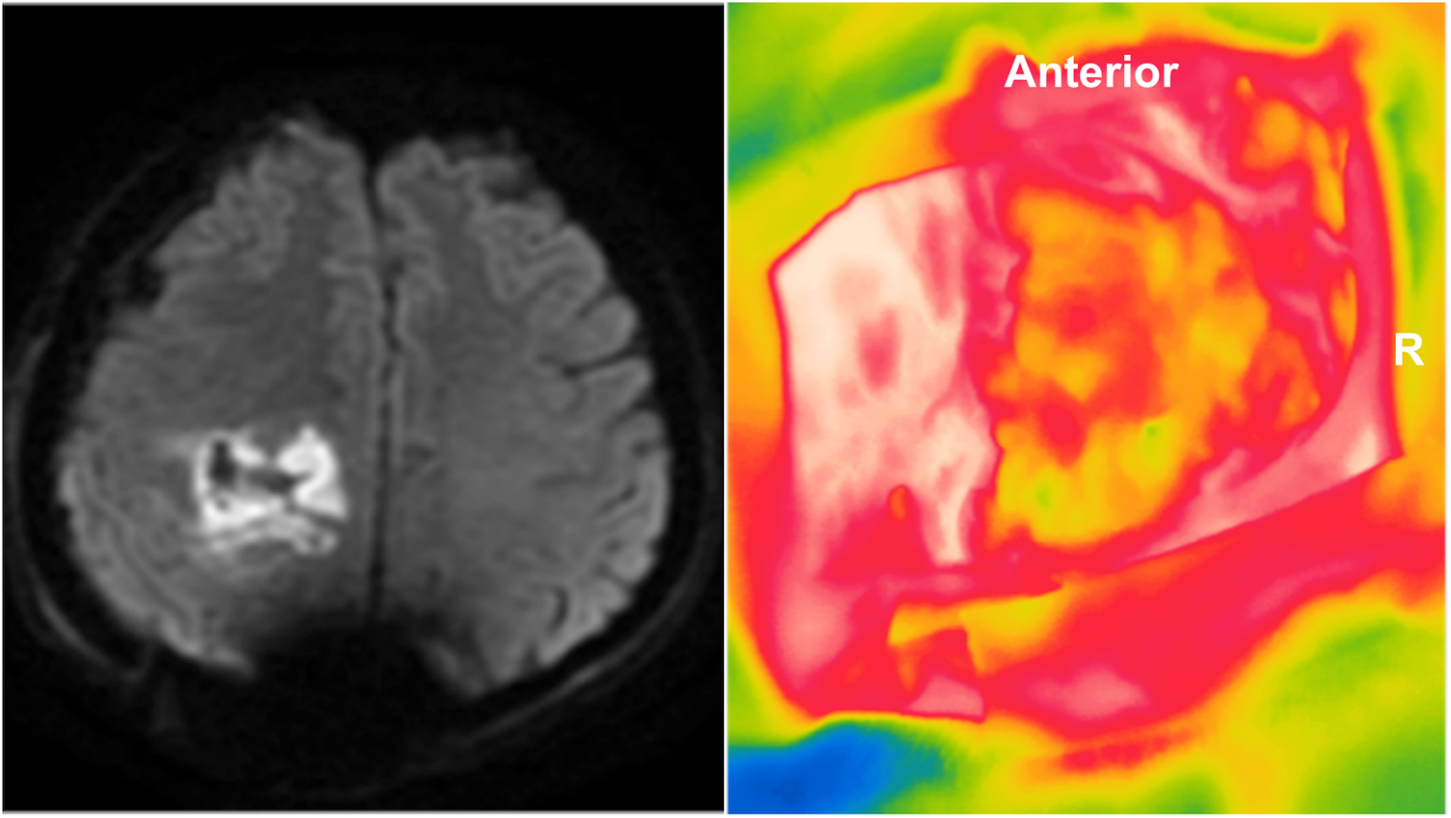
Postoperative MR diffusion-weighted image of a right frontal glioblastoma revealing marked hyperintensity of the tumor bed consistent with acute ischemic injury. Retrospective analysis of the intraoperative infrared thermographic imaging showed that the tumor bed was cooler than the healthy cortex (approximately 2 °C). R, right side.

Seventeen spots (34%) suggested acute cerebral ischemia, based on postoperative diffusion-weighted sequences. Such areas were also retrospectively evaluated revealing a consistent hypothermic map [mean of 1.8 °C (*z* = −4.7827; *p* < 0.0001)] (Figure 5). However, none of the patients presented clinical deterioration.

## 4 Discussion

### 4.1 Main results

The present study included 15 patients with intracranial cortical and subcortical tumors of different histological subtypes. Static and dynamic thermographic maps (cold challenge test) were acquired intraoperatively with a high-resolution camera at pre-established time points. Intraoperative thermographic imaging of the exposed dura mater revealed a mixed vascular pattern of meningeal vessels, veins, and cortical arteries. Intra-axial tumors were reliably identified by demonstrating hypothermic areas, which were exacerbated by the cold challenge test.

Dural opening facilitated both the identification of tumors and their neurovascular relations. The analysis of the C-Spot temperatures significantly elucidated histological subtypes. At the end of the surgical resection, there was a consistent rise in the temperature of gliomas and metastases tumor beds. Residual tumors on imaging were retrospectively evaluated with ITT, presenting a typically delayed temperature recovery curve after the cold challenge test. Acute ischemia was consistently hypothermic.

### 4.2 Interpretation and generalization

#### 4.2.1 Lesion-localization

When performing a craniotomy based on classical craniometric points or even neuronavigation, the surgical team is frequently faced with an imprecise approach or with the difficulty of identifying vascular structures, such as bridging veins and venous sinuses before proceeding with durotomy. It is widely recognized the negative influence of surgical positioning (lateral or prone) is a source of incomplete or inaccurate recordings for navigation (30). Brain shift is the potential main limitation of the method (31, 32). Cerebrospinal fluid drainage, tissue removal, gravity, tumor localization and size, and brain edema are all documented factors that contribute to intraoperative brain deformation (31, 33). The update of the neuronavigation system with iMRI has been a reliable way to compensate for the effects of brain shift (31, 32), but its use is not widespread worldwide, especially for financial restraints.

Intraoperative ultrasound has attracted interest for safety, portability, and real-time imaging (33). In this context, a recent meta-analysis demonstrated a high localization rate (mean of 100%) in identifying the exact location of an intradural tumor (33). Nonetheless, imaging was deemed suboptimal or of poor quality in up to 8% of the patients (33). In addition, image quality and the size of the ultrasound transducer are the main limitations of the method.

Our results demonstrated an elevated ability of ITT for lesion-localization before dural opening (sensitivity and specificity of 93.3% and 100%, respectively), which were confirmed by iUS in some cases and maintenance of surgical strategy for cortical and subcortical tumor resection in all of them. It is worth discussing that for subcortical tumors, ITT mainly detects indirect changes of cortical vascularization caused by tumor necrosis (26), low microvascular density (22, 34), vasogenic edema (35), reduction of cortical metabolism (36), and the occurrence of cystic lesions (25, 27). Such characteristics render typical hypothermic profiles in comparison to the healthy cortex, especially for gliomas. On the other hand, subcortical metastases might occasionally present hyperthermic cortical profiles due to increased vascularization, which was observed in the only false negative patient of our series and a previous study (24). Our surgical strategy was not altered, however, since we could detect the regional changes in the thermographic profile.

Our study introduces a new potential role for ITT with high-resolution cameras in promoting lesion-localization before dural opening, which emerges as an additional method of intraoperative imaging. Gorbach et al. (24) previously tried to promote the identification of intracranial lesions with a medium resolution (256 × 256 pixels) thermographic camera, but they were not successful since the dura mater was identified as a low infrared signal structure, due to evaporative cooling.

#### 4.2.2 Correlation of thermographic and histological profiles

The analysis of thermographic maps by histological type revealed that our results are in agreement with the literature on tumors of glial lineage, given that they were consistently hypothermic, both for cortical tumors or the overlying cortex in subcortical tumors (22–25, 27, 28). The distribution of glial tumors in our sample does not allow investigation of different temperatures by histological subtype, but it is interesting to note that Gorbach et al. (24) reported 10% higher temperatures in oligodendrogliomas WHO grade II in comparison to glioblastomas.

Brain metastases, on the other hand, exhibit a less specific thermal signature depending mostly on the origin of primary cancer. A hypothermic profile is found in lung and breast adenocarcinoma metastases, which are similar to our findings (25, 27), while melanoma metastases usually demonstrate hyperthermic maps (24, 26). Finally, meningiomas mostly demonstrated a hyperthermic profile, especially after dural opening, which has been previously cauterized for adequate devascularization, as also observed by Kastek et al. (25). Gorbach et al. did include one falx meningioma in their series but studied only cortical temperatures, which were also hyperthermic to the surrounding healthy cortex (24). Generally speaking, brain metastases and meningiomas exhibit highly variable thermographic maps that indirectly reveal the grade of tumor vascularization, as well as the presence of edema and cystic degeneration. It should be noted that in meningiomas, the type of vascularization, dural or pial, could also influence the thermographic profile in the case of greater deep nutrition (37).

#### 4.2.3 Infrared intraoperative thermography in the prediction of the extent of resection and acute ischemia

The usefulness of IIT in predicting the extent of resection and acute ischemia has been underestimated in the literature. To the best of our knowledge, only Kateb et al. used IIT for this purpose in a case report (26). In that study, a patient with multiple melanoma brain metastases underwent surgery. A retrospective analysis of IIT after postoperative MRI showed that the residual tumor had a hypothermic tumoral bed.

The occurrence of new postoperative ischemic changes following resection of recently diagnosed and recurrent gliomas, as well as brain metastasis resection is widely recognized and affects approximately 30% of the patients undergoing surgery (38). The rates of transient or new neurological deficits generally did not exceed 10% (38). The detection of new postoperative ischemia, previous radiation therapy, and recurrent tumors conferred higher risks of postoperative neurological deterioration (38). The mechanisms are not fully elucidated, but direct damage by bipolar coagulation and indirect injury by pressure of brain retractors are potential causative factors of ischemic injuries (39, 40).

In an experimental model, Shi and colleagues analyzed the effects of coated and uncoated bipolar coagulation forceps on rat motor cortex. Their thermographic maps revealed extremely high temperatures in both methods, approximately 54 °C, and 64 °C, respectively, at 3 s after coagulation (41). Irrigation was found to be thermo-protective and therefore reduced thermal injury (41).

Our absolute temperature measurements did not reach such a high profile potentially because of the cooling effect of irrigation and the elapsed time before thermographic measurements in two meningiomas with predominant dural feeders (mean increase in temperature of 8 °C). Our findings also demonstrate a less relevant role of static thermographic maps in the detection of residual tumors since tumor beds show a consistent rise in temperature at the end of the procedure. For now, we suggest that this increase results from both the effect of bipolar hemostasis, as described above, and the recovery of cerebral blood flow (24). Thus, the relatively uniform temperature in the tumor bed would make it difficult to identify residual tumors. On the other hand, the thermographic signature created after the cold challenge test proved to be extremely sensitive in the recognition of residual lesions due to the typically delayed temperature recovery curve. Intense hypothermic beds also reflect acute ischemia on postoperative imaging with no clinical consequence, however.

### 4.3 Limitations

The present study has several limitations. First, the limited number of patients precluded the analysis of different thermographic maps in diverse histological subtypes. Second, it is widely recognized that the perilesional cortex exhibits changes in regional cerebral blood flow (24). Thus, it would be possible that the evaluation of thermographic profiles of the contralateral healthy cortex would reveal greater temperature discrepancies. For obvious reasons, we used the temperature of the healthy cortex in the areas exposed by craniotomy as a control. Third, the thermographic camera did not generally have an orthogonal orientation with the surgical bed. Then, some hidden spots could have been missed in the analysis of the extent of resection. Finally, despite postoperative imaging revealing residual tumor tissue, these were not confirmed with tissue biopsy. We are currently running a study to correlate dynamic thermographic maps with residual tumors. Future studies should assess the feasibility of IIT camera integration into the surgical microscope to favor its potential translation into clinical practice and widespread use of this technology in all the issues that we presented.

## 5 Conclusions

High-resolution IIT is a non-invasive alternative intraoperative imaging method for lesion-localization, assessing the extent of tumor resection, and identifying acute ischemia changes with static and dynamic thermographic maps. High-resolution IIT allows the identification of intracranial tumors of up to 2 cm depth with high sensitivity and specificity. Tumors of the glial lineage are consistently hypothermic, while brain metastases and meningiomas exhibit variable thermographic maps. Residual tumors revealed nonspecific static and characteristic dynamic thermographic maps. Acute ischemia was quite frequent, however without clinical consequences.

## Data Availability

The raw data supporting the conclusions of this article will be made available by the authors, without undue reservation.
